# Immunohistochemical Assessment of Immune Response in the Dermis of *Sarcoptes scabiei*—Infested Wild Carnivores (Wolf and Fox) and Ruminants (Chamois and Red Deer)

**DOI:** 10.3390/ani10071146

**Published:** 2020-07-06

**Authors:** Ileana Z. Martínez, Álvaro Oleaga, Irene Sojo, María José García-Iglesias, Claudia Pérez-Martínez, Juan F. García Marín, Ana Balseiro

**Affiliations:** 1 Departamento de Sanidad Animal, Facultad de Veterinaria, Universidad de León, 24006 León, Spain; ileanazorhaya.martinez@upaep.mx (I.Z.M.); isojoa00@estudiantes.unileon.es (I.S.); mjgari@unileon.es (M.J.G.-I.); cperm@unileon.es (C.P.-M.); jfgarm@unileon.es (J.F.G.M.); 2 Universidad Popular Autónoma del Estado de Puebla, UPAEP Universidad, 72410 Puebla, Mexico; 3 SERPA, Sociedad de Servicios del Principado de Asturias S.A., 33202 Gijón, Spain; alvaroleaga@yahoo.es; 4 Departamento de Sanidad Animal, Instituto de Ganadería de Montaña, CSIC-Universidad de León, Finca Marzanas, Grulleros, 24346 León, Spain

**Keywords:** *Sarcoptes scabiei*, dermis cellular response, wolf, red fox, chamois, red deer, immunohistochemistry

## Abstract

**Simple Summary:**

This article studies the local immune processes in dermis underlying the macroscopical differences (hyperkeratotic or alopecic) in mangy lesions from wolves (*Canis lupus*), foxes (*Vulpes vulpes*), chamois (*Rupicapra rupicapra*) and red deer (*Cervus elaphus*) naturally infested with *Sarcoptes scabiei*. Skin sections were immuno-stained to detect macrophages, plasma cells, T lymphocytes and B lymphocytes. Skin lesions contained significantly more inflammatory cells in fox than in wolf and chamois. Macrophages were the most abundant inflammatory cells in the lesions of all the species studied, suggesting a predominantly innate, non-specific immune response. Lesions from wolf contained higher proportions of macrophages than the other species, which may reflect a more effective response, leading to alopecic lesions. Fox and chamois may also mount substantial humoral and cellular immune responses with apparently scarce effectiveness that lead to hyperkeratotic lesions.

**Abstract:**

Sarcoptic mange is caused by the mite *Sarcoptes scabiei* and has been described in several species of domestic and wild mammals. Macroscopic lesions are predominantly hyperkeratotic (type I hypersensitivity) in fox, chamois and deer, but alopecic (type IV hypersensitivity) in wolf and some fox populations. To begin to understand the immune processes underlying these species differences in lesions, we examined skin biopsies from wolves (*Canis lupus*), foxes (*Vulpes vulpes*), chamois (*Rupicapra rupicapra*) and red deer (*Cervus elaphus*) naturally infested with *S. scabiei*. Twenty skin samples from five animals per species were used. Sections were immuno-stained with primary antibodies against Iba1 to detect macrophages, lambda chain to detect plasma cells, CD3 to detect T lymphocytes and CD20 to detect B lymphocytes. Skin lesions contained significantly more inflammatory cells in the fox than in the wolf and chamois. Macrophages were the most abundant inflammatory cells in the lesions of all the species studied, suggesting a predominantly innate, non-specific immune response. Lesions from the wolf contained higher proportions of macrophages than the other species, which may reflect a more effective response, leading to alopecic lesions. In red deer, macrophages were significantly more abundant than plasma cells, T lymphocytes and B lymphocytes, which were similarly abundant. The fox proportion of plasma cells was significantly higher than those of T and B lymphocytes. In chamois, T lymphocytes were more abundant than B lymphocytes and plasma cells, although the differences were significant only in the case of macrophages. These results suggest that all the species examined mount a predominantly innate immune response against *S. scabiei* infestation, while fox and chamois may also mount substantial humoral and cellular immune responses, respectively, with apparently scarce effectiveness that lead to hyperkeratotic lesions.

## 1. Introduction

Sarcoptic mange is a parasitic skin disease that has been described in several species of domestic and wild mammals, and it is also a zoonosis that affects humans. It is caused by the burrowing mite *Sarcoptes scabiei*, which has caused epizootics involving high morbidity and mortality rates in some wild mammal populations, mainly ungulate species [1,2]. Different host species, and even individuals within the same species, show variations in the severity and location of the lesions. For example, studies in the Southern UK [3] and Northern Spain [4,5] have reported that the disease manifests predominantly as hyperkeratotic lesions (type I hypersensitivity) in wild ungulates such as chamois and red deer, but as alopecic lesions (type IV hypersensitivity) in the wolf and fox. Nevertheless, hyperkeratotic lesions are also commonly observed in foxes [3,5,6].

Species and individual differences have been attributed to differences in the immune response [7], clinical stage [6,8] and concomitant presence of immunosuppressive pathogens [9]. In fact, *S. scabiei* itself can modulate various aspects of the mammalian innate and adaptive immune responses [10]. Identifying what immune responses are triggered upon infestation with *S. scabiei* and their relative efficacy may improve our understanding of disease course. The most common immune response in ectoparasite-associated systemic or local dermatitis in response to mites and its products is recruitment of eosinophils together with mast cells, typical of type I hypersensitivity [11]. Langerhans cells in the epidermis internalize sarcoptic antigen and migrate to regional lymph nodes, where they stimulate T cells [12]. In humans, the combined action of macrophages, T lymphocytes, and eosinophils can limit mite numbers and lesions [13]. Other inflammatory cells observed in mange lesions are plasma cells and B lymphocytes, which produce immunoglobulins that participate in the humoral immune response [14,15].

The main goal of this study was to analyze relative proportions of macrophages, plasma cells, as well as T and B lymphocytes in the skin of the wolf, fox, chamois and red deer from Asturias (Northern Spain) that were naturally infested with *S. scabiei*. The results may help us understand why infestation leads to different skin lesion patterns in certain species, which in turn may help guide efforts to manage the disease, i.e., treatment following capture in the wild of most susceptible species.

## 2. Materials and Methods

### 2.1. Samples

In previous work skin samples were taken from two wild carnivore species, the wolf (*Canis lupus)* and fox (*Vulpes vulpes*), and from two wild ruminant species, chamois (*Rupicapra rupicapra)* and red deer (*Cervus elaphus*), all from Asturias (Northern Spain) [5]. Afterwards, skin samples were paraffin-embedded, stained with hematoxylin–eosin and found to have natural sarcoptic mange, which was confirmed by mite isolation and identification. Gross examination showed that the wolf lesions were alopecic, while the fox, chamois and red deer lesions were extensive and hyperkeratotic. *Sarcoptes scabiei* mite burden was high in all species except the wolf. Eosinophils were not observed in wolf skin samples but they were observed in the other species [5]. Mast cells were not observed in any species.

Skin samples from 20 animals (5 per species) were selected for immunohistochemistry in this work. Ethical permission was not required.

### 2.2. Immunohistochemistry

Serial 3-µm paraffin sections were used for immunohistochemical detection of four different antigens (Table 1) using the Avidin-Biotin Complex (ABC) method (Vector Laboratories, CA, USA). Briefly, the sections were deparaffinized, rehydrated and rinsed with tap water. Endogenous peroxidase was quenched by incubating sections in methanol containing 3% H_2_O_2_ for 10 min at room temperature, then they were washed with water for 10 min. Antigens were retrieved using epitope demasking (Table 1), and nonspecific binding was inhibited by incubating the sections for 20 min at room temperature with 10% normal horse serum (detection of CD3) or 10% normal goat serum (detection of Iba1, lambda chain or CD20) in Tris-buffered saline (TBS) containing 5 mM Tris•HCl (pH 7.6), 136 mM NaCl and 1% bovine serum albumin. Tissue sections were incubated overnight at 4 °C with commercial mono- or polyclonal primary antibodies (Table 1), and then washed three times with TBS. Samples were incubated for 30 min at room temperature with horse anti-mouse serum or goat anti-rabbit serum (1:200, Vector Laboratories; Table 1), washed three times with TBS and then incubated with the ABC kit in TBS for 30 min at room temperature.

Finally, the sections were incubated for 5 min with the substrate 3,3’-diaminobenzidine tetrahydrochloride (DAB; Sigma, St. Louis, MO, USA) and washed with TBS and water. After staining for 45 s with hematoxylin, slides were dehydrated and mounted with DPX (Fluka, Sigma, St. Louis, MO, USA). Stained slides were studied under light microscopy (Olympus BH—2) and photographed using a digital camera (Olympus DP—12). Each immunohistochemical staining included a positive control, in which the target antigen was present in the control section and the specific antibody was used (Figure S1); as well as a negative control, in which the primary antibody was omitted.

### 2.3. Cell Counting and Statistical Analysis

A total of 80 slides (4 slides per animal, 5 animals per species) were used for cell counting. Cells positive for each immunostained marker were quantified in five fields of each slide at 400× magnification using an image analysis program (Imaging Software NIS-Elements 3.20, Nikon, Tokyo, Japan). Then the mean proportion of stained cells to total cells was averaged across the five fields.

Descriptive and inferential statistics were used to analyze the distribution of four types of inflammatory cells in skin mange lesions in the four species. Data were tested for normality using the Shapiro–Wilk test. Species differences in the total number of inflammatory cells in scabies skin lesions were assessed for significance using the non-parametric Kruskal–Wallis and pairwise comparison tests. The percentage of total cells that stained for each of the four inflammatory cell biomarkers (Iba1, lambda chain, CD3 and CD20) was compared within and between each species using a one-way ANOVA. When significant differences were found, the Tukey test for multiple comparisons was applied.

As appropriate, data were expressed as the mean and standard deviation, or as the median and interquartile range. Data were analyzed using SPSS 24 for Windows (IBM, Chicago, IL, USA). A significance level of 0.05 was applied.

## 3. Results

### 3.1. Total Number of Inflammatory Cells

Samples from all species showed high numbers of inflammatory cells, with the fox showing the highest number. Inflammatory infiltrate was significantly higher in the fox than in the wolf and chamois (Table 2). On the other hand, while intra-species variation in the number of inflammatory cells was low for the fox and red deer, two of the five wolf samples showed lower numbers, and one chamois sample showed a much higher number.

### 3.2. Relative Proportions of Inflammatory Cell Types within Each Species

**Figure 1:**
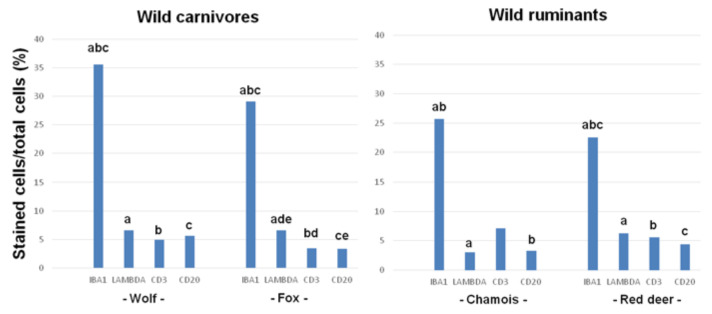
Intra-species differences in the numbers of macrophages (based on Iba1 immunostaining), plasma cells (lambda chain), T lymphocytes (CD3) and B lymphocytes (CD20) in skin mange lesions from wild carnivores and ruminants. For each animal species, the same letters above different rectangular bars indicates significant differences between means. For wolf: (a, b, c) *p* < 0.001. For fox: (a, b, c, d, e) *p* < 0.05. For chamois: (a, b) *p* < 0.05. For red deer: (a, b, c) *p* < 0.001. A Tukey test for multiple comparisons was applied for statistical analysis.

In all species, the most abundant cells in the inflammatory infiltrate were macrophages, while the least abundant were T or B lymphocytes (Figure 1).

**Figure 2:**
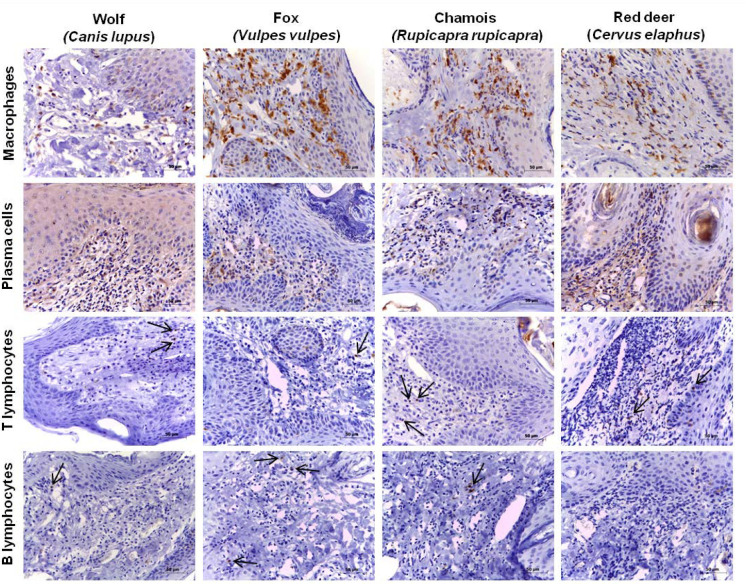
Comparative immunohistochemistry of cellular response in the dermis of wolves, foxes, chamois and red deer naturally infested with *Sarcoptes scabiei*. Skin biopsies were stained with primary antibodies against the indicated markers and the avidin-biotin complex kit. Macrophages were the predominant type of inflammatory cell in all species (top row). Plasma cells were present in all species, but less abundant than macrophages (second row). T lymphocytes (third row) and B lymphocytes (bottom row) were scarce in all species. Arrows indicate examples of stained cells when they are scarce (even with no B lymphocytes in red deer). Tissue sections in each column came from the same animal, and the results shown are representative of all five animals of each species. Bars = 50 microns.

In the wolf and red deer, macrophages were significantly more abundant than plasma cells, T lymphocytes and B lymphocytes, which were similarly abundant (Figure 1 and Figure 2). In the fox, the proportion of macrophages was significantly higher than the proportions of other cell types, and the proportion of plasma cells was significantly higher than those of T and B lymphocytes (Figure 1). In chamois, macrophages and T lymphocytes were more abundant than B lymphocytes and plasma cells (Figure 1 and Figure 2), although the differences were significant only in the case of macrophages.

### 3.3. Relative Proportions of Inflammatory Cell Types across Species

Wolves showed more abundant macrophages than the other species, although the difference was significant only with red deer (Table 3). Plasma cells were similarly abundant in the wolf, fox and red deer; the abundance in the wolf and fox was significantly higher than in chamois. Abundance of T and B lymphocytes was similarly low across all four species. Of all species, chamois showed the highest abundance of T lymphocytes and wolf showed the highest abundance of B lymphocytes, though the differences across the four species were not significant.

## 4. Discussion

This study examined the immune response in the dermis of the wolf, fox, chamois and red deer from Northern Spain against natural *S. scabiei* infestation. We identified macrophages as the predominant cells in lesions of all four species, and we found small differences in the immune response among species.

Our observation that fox lesions contained the highest number of inflammatory cells may mean that the animals were in a more severe stage of the disease, or it may mean that their immune response of macrophages and lymphocytes was unable to control disease progression based on the generalized and crust lesions observed in those animals [6,8]. We cannot exclude that the higher cell number reflects higher parasitic burden [5], but this seems less likely, since chamois and red deer showed less intense inflammatory response despite high parasite burden [5]. Instead, the less intense response in the wolf, chamois and red deer may reflect some kind of adaptation or tolerance between the host and parasite [6,16]. These results should be verified and extended in further studies, especially since they varied appreciably among species and individuals within each species.

In all four species, macrophages were the most abundant inflammatory cells in skin lesions, yet the proportions varied across species. Lesions from the wolf showed a higher percentage of macrophages than lesions from other species, while lesions from fox and chamois showed a higher percentage of plasma cells and T lymphocytes, respectively. This may help explain species differences in hypersensitivity responses [5,16]. Ungulates and some fox populations tend to show immediate type I hypersensitivity response with higher eosinophil counts, while the wolf and other fox populations tend to show delayed type IV hypersensitivity response with higher macrophage counts [5]. Higher macrophage count may help explain the greater efficacy of a type IV hypersensitivity (alopecic) response in the wolf for eliminating *S. scabiei* [5]. The lower percentage of macrophages in foxes, chamois and red deer in the present study, together with the higher number of eosinophils previously observed in mange lesions [5], may result primarily in hyperkeratotic lesions related to a late phase of the type I hypersensitivity response pointing towards type IV hypersensitivity [6].

The higher proportion of plasma cells in fox than those of T and B lymphocytes might suggest a stronger humoral response [17]. In contrast, chamois in our study showed a larger proportion of T lymphocytes than B lymphocytes and plasma cells, consistent with previous work [8], confirming that in this species the cellular immune response is much stronger than the humoral response to *S. scabei* infestation [17]. That response may be related to the severe hyperkeratotic type lesions in our animals, which might reflect the ineffective T helper 2 (Th2) lymphocyte-type immune response [8]. In fact, excessive signaling by Th2 lymphocytes can trigger atopies or allergies [12]. The relative proportions of the different types of inflammatory cells may translate to differences in immune efficacy against *S. scabiei* infestation. Future studies should explore the factors that influence the nature and efficacy of the immune response against *S. scabiei*, which likely include body condition, sex, age, clinical stage, concomitant presence of immunosuppressive pathogens and sampling season [9]. Our results illustrate how immunohistochemistry of skin samples from wild species affected by sarcoptic mange can be useful for analyzing the immune response to infestation.

## 5. Conclusions

Our studies of four host species indicated a low proportion of B lymphocytes, T lymphocytes and plasma cells and a high proportion of macrophages in response to *S. scabiei* infestation, suggesting that these species mount a primarily innate immune response and are relatively poor at developing an adaptive immune response to this pathogen. Our findings further suggest that sarcoptic mange skin lesions may reflect a substantial humoral immune response in fox or a cellular immune response in chamois. Our observation of highest macrophage abundance in wolves may help explain their apparently more effective immune response against *S. scabiei.*

## Figures and Tables

**Table 1:** Immunohistochemical protocols used to characterize types of inflammatory cell in skin lesion biopsies.

Primary Antibody (Dilution)	Target Cell Type	Epitope Demasking	Biotinylated Secondary Antibody (Dilution)
Iba1 (WAKO 019_19741), rabbit polyclonal (1:1000)	Macrophage	Microwave in citrate (pH 6), 20 min	Anti-rabbit (1:200)
Lambda (Dako A0193), rabbit polyclonal (1:1000)	Plasma cell	Microwave in citrate (pH 6), 20 min	Anti-rabbit (1:200)
**CD3 (Novocastra-CL-L-CD3-565), mouse monoclonal (1:500)**	pan-T cell	Microwave in citrate (pH 6), 20 min	Anti-mouse (1:200)
CD20 (ThermoFisher-PA516701), rabbit polyclonal (1:200)	pan-B cell	Steamer in citrate (pH 6), 20 min	Anti-rabbit (1:200)

**Table 2:** Total numbers of inflammatory cells in skin mange lesions from four species.

Species	n	Mean ± SD *	Median	IQR
Wolf	5	1175.2 ± 135.9 ^a^	1179	1044.0–1304.5
Fox	5	1636.4 ± 195.8 ^ab^	1725	1431.5–1797.0
Chamois	5	1242.4 ± 232.4 ^b^	1192	1088.5–1421.5
Red deer	5	1293.6 ± 137.3	1228	1206.5–1413.5

* SD, standard deviation. IQR, interquartile range. ^a,b^ Statistical analysis by pairwise comparisons showed significant differences between means followed by same letters in the same column (*p* < 0.05).

**Table 3:** Percentages of cells staining positive for inflammatory cell biomarkers in skin lesions from four species.

Biomarker (Target Cell Type)	Animal Species	% Positive Cells		
		n	Mean *	SD
Iba1 (macrophages)	Wolf	5	35.59 ^a^	4.11
	Fox	5	29.11	7.94
	Chamois	5	25.70	9.60
	Red deer	5	22.57 ^a^	3.96
Lambda chain (plasma cells)	Wolf	5	6.61 ^b^	0.97
	Fox	5	6.56 ^c^	1.29
	Chamois	5	2.99 ^bc^	0.28
	Red deer	5	6.31	3.19
CD3 (T lymphocytes)	Wolf	5	4.88	1.88
	Fox	5	3.39	1.53
	Chamois	5	7.15	3.59
	Red deer	5	5.59	1.49
CD20 (B lymphocytes)	Wolf	5	5.60	2.08
	Fox	5	3.33	0.90
	Chamois	5	3.31	0.93
	Red deer	5	4.42	1.87

* SD, standard deviation. Means followed by same letters in the same column differ significantly (Tukey test): ^a^ *p* = 0.038, ^b^ *p* = 0.004 and ^c^ *p* = 0.017.

## Supplementary Materials

The following are available online at https://www.mdpi.com/2076-2615/10/7/1146/s1, Figure S1: Immunohistochemical technique in positive controls; lymph node from badger.
