# Supplementary figures and images for: Immunohistochemical Assessment of Immune Response in the Dermis of Sarcoptes scabiei—Infested Wild Carnivores (Wolf and Fox) and Ruminants (Chamois and Red Deer)
Source: Animals (Basel). 2020 Jul 6;10(7):1146. doi: 10.3390/ani10071146 (PMC7401513; doi:10.3390/ani10071146)

Macrophages

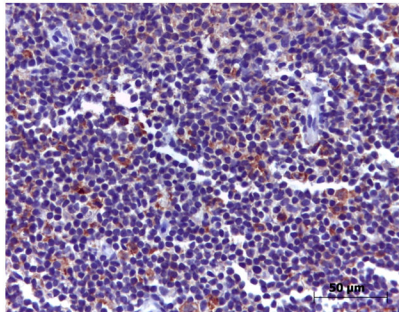

Plasma cells

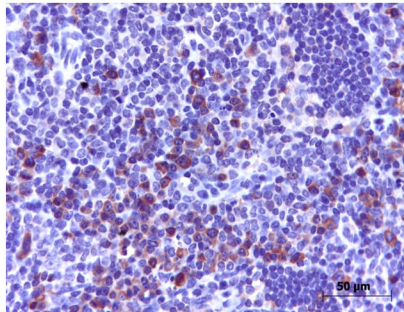

T lymphocytes

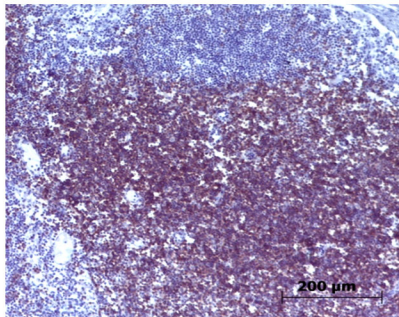

B lymphocytes

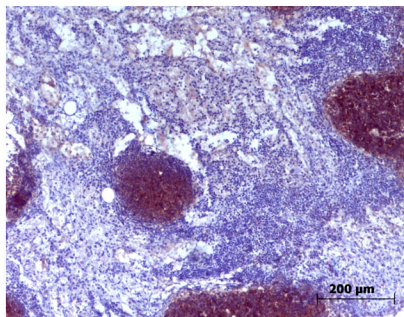

Supplement: Supplementary file 1 [file animals-10-01146-s001.pdf]
